# Crystal structure of l-leucyl-l-isoleucine 2,2,2-tri­fluoro­ethanol monosolvate

**DOI:** 10.1107/S2056989016005302

**Published:** 2016-04-05

**Authors:** Carl Henrik Görbitz

**Affiliations:** a Department of Chemistry, University of Oslo, PO Box 1033 Blindern, N-0315 Oslo, Norway

**Keywords:** dipeptide, head-to-tail chains, hydrogen bonds, layered structure, crystal structure

## Abstract

Hydro­phobic dipeptides with either l-Leu or l-Phe constitute a rather heterogeneous group of crystal structures. Some form materials with large water-filled channels, but there is also a pronounced tendency to incorporate organic solvent mol­ecules, which then act as acceptors for one of the three H atoms of the charged N-terminal amino group. l-Leu-l-Ile has uniquely been obtained as two distinct hydrates, but has so far failed to co-crystallize with a simple alcohol. The present structure of C_12_H_24_N_2_O_3_·CF_3_CH_2_OH, which crystallizes with two dipeptide and two solvent mol­ecules in the asymmetric unit, demonstrates that when 2,2,2-tri­fluoro­ethanol is used as a solvent, its high capacity as a hydrogen-bond donor leads to formation of an alcohol solvate.

## Abstract 2

Unlike several other dipeptides with two hydro­phobic residues, l-Leu-l-Ile has not previously been obtained as an alcohol solvate, forming instead two different hydrates. Formation of a co-crystal has here been achieved by using a 2,2,2-tri­fluoro­ethanol solution. As expected, the resulting structure is divided into hydro­philic and hydro­phobic layers.

## Chemical context   

Dipeptides with at least one hydro­phobic residue (*i.e.* lacking a functional group) such as Val, Leu, Ile and Phe have a high propensity to form crystal structures that are divided into hydro­phobic and hydro­philic layers (Görbitz, 2010 ▸). The latter include two *C*(8) head-to-tail chains with two of the three N-terminal amino H atoms acting as donors and the C-terminal carboxyl­ate group as acceptor, and also a *C*(4) or *C*(5) chain using the peptide >N—H group as donor and, respectively, the peptide carbonyl group or the carboxyl­ate group as acceptor. The third amino H atom finds an acceptor in a polar side chain or, when both residues are hydro­phobic, in a co-crystallized solvent mol­ecule. l-Leu-l-Val has thus been obtained as a series of alcohol solvates (Görbitz & Torgersen, 1999 ▸), but also as a non-layered hydrate (Görbitz & Gundersen, 1996 ▸). The same is true for l-Leu-l-Leu (Görbitz, 1998 ▸, 2001 ▸). l-Leu-l-Ile (LI) has, on the other hand, been obtained as two distinct hydrates; a 0.75 hydrate (Görbitz, 2004 ▸; CSD refcode ETIWIN) that is isostructural to the Leu-Val analogue (Görbitz & Gundersen, 1996 ▸), and a 2.5 hydrate with extensive water channels (Görbitz & Rise, 2008 ▸; CSD refcode HIZCOJ). Crystallization using methanol, ethanol or 2-propanol as precipitating agents did not result in formations of alcohol solvates.
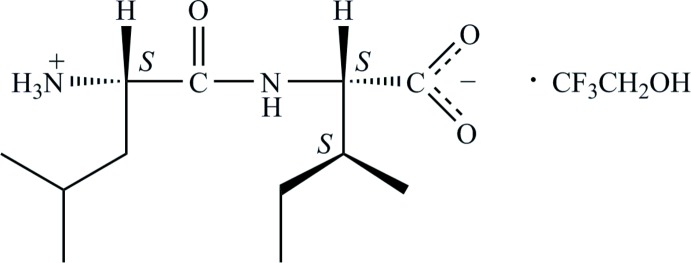


Recently we have become inter­ested in the use of fluorinated alcohols like 2,2,2-tri­fluoro­ethanol (TFE) and 1,1,1,3,3,3-hexa­fluoro-2-propanol during crystallization, not only due to their superior abilities to dissolve a large range of organic mol­ecules (abandoning the use of water if that is desirable), but also as crystal engineering tools to manipulate hydrogen-bonding patterns in solid-state structures by being incorporated into the crystal lattice by virtue of their strong hydrogen-bond-donating capacity. The crystal structure of the LI TFE solvate (I) presented here provides an example of how this can take place.

## Structural commentary   

**Figure 1:**
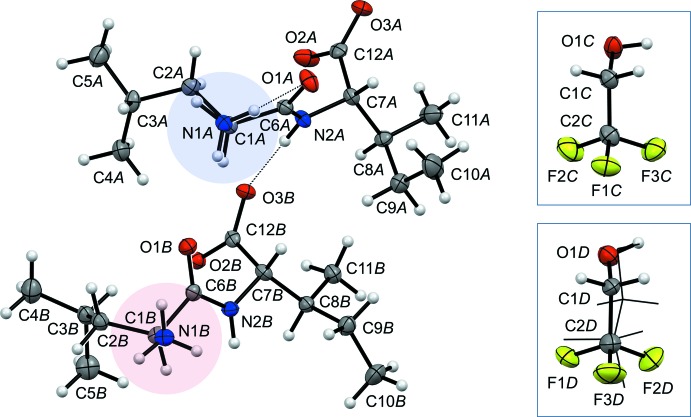
The asymmetric unit of (I), solvent mol­ecules being shown in different positions relative to the peptide mol­ecules than they have in the unit cell to avoid extensive overlap. The minor disorder orientation for TFE mol­ecule *D* is shown in wireframe representation. The amino group of mol­ecule *A* has an unusual eclipsed conformation (blue shade) resulting from formation of an intra­molecular hydrogen bond to O1*A*, while a normal staggered conformation (red shade) is observed for mol­ecule *B*. Thermal displacement ellipsoids are shown at the 50% probability level.

**Figure 2:**
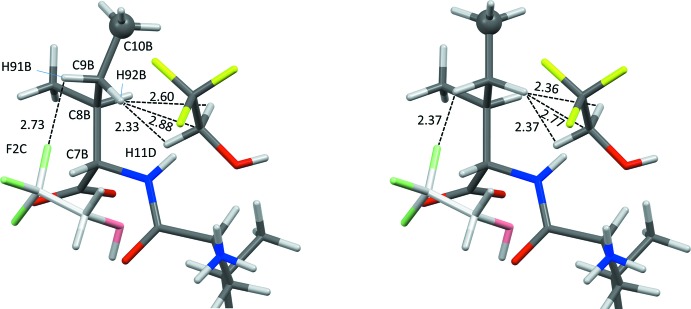
In the experimental crystal structure of (I) (left) the ethyl group of the Ile residue of mol­ecule *B* is rotated to relieve a short distance between H91*B* and F2*C*. If the C7*B*—C8*B*—C9*B*—C10*B* torsion angle had been exactly 180°, this distance would have been too short (right). The terminal methyl group, with C10*B* as a sphere, is not involved in any short contacts.

The four mol­ecules (two dipeptides and two solvent species) in the asymmetric unit are shown in Fig. 1 ▸. The structure is well behaved with normal bond lengths and bond angles. Disorder for TFE mol­ecule *D* was easily resolved (see *Refinement details*). The mol­ecular conformations of the two peptide mol­ecules are quite different in terms of the side-chain conformations, Table 1 ▸. The overall mol­ecular conformation of mol­ecule *B* is very close to that of mol­ecule *B* in the 2.5 hydrate (Görbitz & Rise, 2008 ▸). A substantial 24.5° deviation from the idealized *trans* orientation at 180° for χ_2_^2^ of mol­ecule *B* is needed to relieve a short contact between H91*B* and F2*C*, Fig. 2 ▸.

## Supra­molecular features   

**Figure 3:**
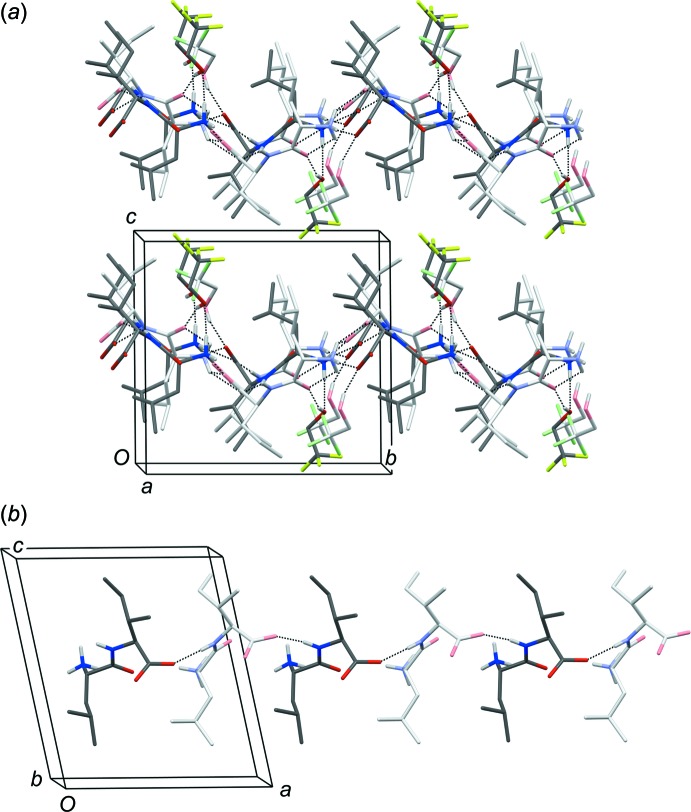
Crystal packing of (I) viewed (*a*) approximately along the *x* axis. (*b*) View approximately along the *y* axis showing a single hydrogen-bonded *C*(5) chain parallel to the *x* axis. Only H atoms involved in strong hydrogen bonds are included; peptide mol­ecule *A* and TFE mol­ecule *C* are shown with atoms in lighter colors.

The unit cell and crystal-packing arrangement is illustrated in Fig. 3 ▸ *a*), hydrogen-bond parameters are listed in Table 2 ▸. While the two mol­ecules in the asymmetric unit of structures like l-Met-l-Ala 2-propanol solvate (Görbitz, 2000 ▸; CSD refcode CAQTOD) and l-Leu-l-Phe 2-propanol solvate (Görbitz, 1999 ▸; CSD refcode COCGOQ) are quite similar and related by pseudotranslational symmetry along a 10 Å long axis, the differences between the conformations (as discussed above) and relative positions of LI mol­ecules *A* and *B* are readily observed in Fig. 3 ▸ *b*). The *C*(5) hydrogen-bonded chain is part of an ***S*** **5** hydrogen-bonded sheet, one out of four distinct types of sheets observed in layered dipeptide crystal structures (Görbitz, 2010 ▸).

**Figure 4:**
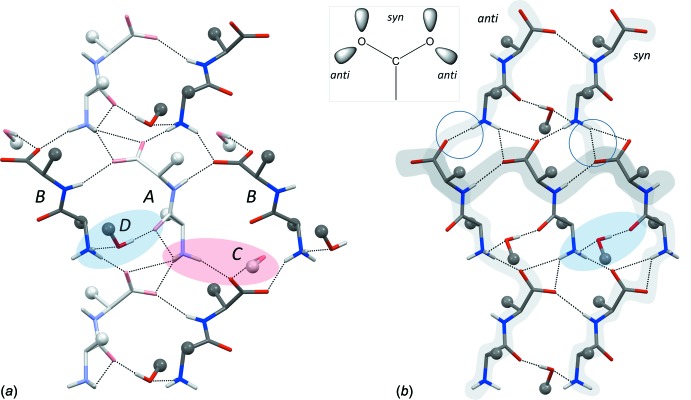
Hydrogen bonds in (*a*) the crystal structure of (I) and (*b*) the crystal structure of l-Leu-l-Val 2-propanol solvate (Görbitz & Torgersen, 1999 ▸; CSD refcode JUCSEF01). Peptide Cβ atoms and solvent C atoms carrying hydroxyl groups are shown as small spheres, other side-chain and solvent atoms have been omitted for clarity. The archetype ***S*** **5** pattern in (*b*) is characterized by the presence of one *syn* and one *anti* head-to-tail *C*(8) chain with alternating mol­ecules being related by ***S***crew symmetry (light grey shades), as well as a *C*(**5**) chain involving an amide >N—H donor and a carboxyl­ate acceptor. An ***S*** **4** pattern has the same symmetry, but a *C*(**4**) chain to O=C< carbonyl acceptor, while consecutive mol­ecules in ***T*** **5** and ***T*** **4** sheets are related by ***T***ranslation rather than by a screw operation (Görbitz, 2010 ▸). See text for details on the red and blue shades.

This sheet is compared in Fig. 4 ▸ to the corresponding sheet of l-Leu-l-Val 2-propanol solvate (Görbitz & Torgersen, 1999 ▸), where the third amino hydrogen atom is accepted by the co-crystallized alcohol mol­ecule (shaded blue in Fig. 4 ▸ *b*). At the same time, the hydroxyl group serves as a hydrogen-bond donor to the peptide carbonyl group, which is not involved in any other strong hydrogen bonds (in distinction to the related ***S*** **4** pattern). Precisely the same function is taken by TFE mol­ecule *D* in Fig. 4 ▸ *a*), but solvent mol­ecule *C* is different; it seeks out and forms a hydrogen bond to the carboxyl­ate group of peptide mol­ecule *B*, uniquely abandoning its role as a hydrogen-bond acceptor (red shade in Fig. 4 ▸ *b*). The third amino H atom of mol­ecule *A* is then left to participate in only a bent intra­molecular inter­action that leads to the inherently less favorable eclipsed amino conformation shown in Fig. 1 ▸.

In summary, TFE has been shown to be co-crystallized with L-Leu-L-Ile, thus radically changing the hydrogen bonding pattern. Is is the first dipeptide alcohol solvate where an alcohol mol­ecule does not act as a hydrogen bond acceptor, but rather forms a strong hydrogen bond donor to a peptide carboxyl­ate acceptor.

## Synthesis and crystallization   

l-Leu-l-Val was purchased from Sigma–Aldrich and used as received. Colorless plates of the title compound were grown by vapor diffusion of aceto­nitrile into 30 µl of a saturated tri­fluoro­ethanol solution of the dipeptide.

## Refinement details   

Crystal data, data collection and structure refinement details are summarized in Table 3 ▸. Solvent mol­ecule *D* is disordered over a major and a minor position with occupancies 0.825 (5) and 0.175 (5), respectively. The O1 and C1 atoms of the minor component were constrained to have the same set of anisotropic displacement parameters as the corresponding atoms of the major component, while C2 and the three F atoms were refined isotropically.

## Supplementary Material

Crystal structure: contains datablock(s) I. DOI: 10.1107/S2056989016005302/hb7570sup1.cif

Structure factors: contains datablock(s) I. DOI: 10.1107/S2056989016005302/hb7570Isup2.hkl

Supporting information file. DOI: 10.1107/S2056989016005302/hb7570Isup3.cml

CCDC reference: 1471080

Additional supporting information:  crystallographic information; 3D view; checkCIF report

## Figures and Tables

**Table 1:** Selected torsion angles (°)

Torsion angle	Name	Mol­ecule *A*/Mol­ecule *B*	Conformation *A*/*B*
N1—C1—C6—N2	ψ_1_	162.6 (3)/117.8 (3)	–/–
C1—C6—N2—C7	ω_1_	168.6 (3)/173.1 (3)	–/–
C6—N2—C7—C12	φ_2_	−99.6 (4)/−65.5 (4)	–/–
N2—C7—C12—O2	ψ_T_	−52.8 (4)/−41.1 (4)	–/–
N1—C1—C2—C3	χ_1_^1^	−69.5 (4)/177.8 (3)	*gauche*−/*trans*
C1—C2—C3—C4	χ_1_^2,1^	−68.2 (4)/−168.3 (3)	*gauche*−/*trans*
C1—C2—C3—C5	χ_1_^2,2^	170.1 (3)/69.1 (4)	*trans*/*gauche*+
N2—C7—C8—C9	χ_2_^1,1^	−60.9 (4)/−72.7 (3)	*gauche*−/*gauche*−
N2—C7—C8—C11	χ_2_^1,2^	173.8 (3)/161.1 (3)	*trans*/*trans*
C7—C8—C9—C10	χ_2_^2^	−59.5 (4)/155.5 (3)	*gauche*−/*trans*

**Table 2:** Hydrogen-bond geometry (Å, °)

*D*—H⋯*A*	*D*—H	H⋯*A*	*D*⋯*A*	*D*—H⋯*A*
N1*A*—H1*A*⋯O3*A* ^i^	0.91	2.13	2.928 (4)	146
N1*A*—H2*A*⋯O1*A*	0.91	2.07	2.607 (4)	116
N1*A*—H3*A*⋯O2*B* ^ii^	0.91	1.87	2.767 (4)	168
N2*A*—H4*A*⋯O3*B*	0.88	2.00	2.883 (4)	177
N1*B*—H1*B*⋯O2*A* ^ii^	0.91	1.79	2.695 (4)	179
N1*B*—H2*B*⋯O3*B* ^ii^	0.91	1.89	2.721 (4)	151
N1*B*—H3*B*⋯O1*D*	0.91	1.98	2.838 (5)	156
O1*D*—H1*D*⋯O1*A* ^iii^	0.86 (3)	1.87 (4)	2.695 (4)	159 (5)
O1*C*—H1*C*⋯O2*B* ^ii^	0.86 (3)	1.85 (3)	2.693 (4)	167 (4)

Symmetry codes: (i) 
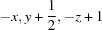
; (ii) 
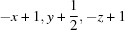
; (iii) 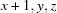.

**Table 3:** Experimental details

Crystal data	
Chemical formula	C_12_H_24_N_2_O_3_·C_2_H_3_F_3_O
*M*_r_	344.37
Crystal system, space group	Monoclinic, *P*2_1_
Temperature (K)	120
*a*, *b*, *c* (Å)	10.947 (3), 12.999 (4), 12.440 (4)
β (°)	101.833 (4)
*V* (Å^3^)	1732.6 (9)
*Z*	4
Radiation type	Mo *K*α
μ (mm^−1^)	0.12
Crystal size (mm)	0.77 × 0.43 × 0.07

Data collection	
Diffractometer	Bruker D8 Advance single crystal CCD
Absorption correction	Multi-scan (*SADABS*; Bruker, 2014 ▸)
*T*_min_, *T*_max_	0.643, 1.000
No. of measured, independent and observed [*I* > 2σ(*I*)] reflections	10435, 5594, 4796
*R*_int_	0.039
(sin θ/λ)_max_ (Å^−1^)	0.598

Refinement	
*R*[*F* ^2^ > 2σ(*F* ^2^)], *wR*(*F* ^2^), *S*	0.041, 0.095, 1.03
No. of reflections	5594
No. of parameters	454
No. of restraints	39
H-atom treatment	H atoms treated by a mixture of independent and constrained refinement
Δρ_max_, Δρ_min_ (e Å^−3^)	0.27, −0.18

Computer programs: *APEX2* and *SAINT-Plus* (Bruker, 2014 ▸), *SHELXT* (Sheldrick, 2015*a* ▸), *Mercury* (Macrae *et al.*, 2008 ▸) and *SHELXL2014* (Sheldrick, 2015*b* ▸).

## supplementary crystallographic information

### Crystal data

**Table:**

C_12_H_24_N_2_O_3_·C_2_H_3_F_3_O	*F*(000) = 736
*M**_r_* = 344.37	*D*_x_ = 1.320 Mg m^−^^3^
Monoclinic, *P*2_1_	Mo *K*α radiation, λ = 0.71073 Å
*a* = 10.947 (3) Å	Cell parameters from 4820 reflections
*b* = 12.999 (4) Å	θ = 2.3–25.0°
*c* = 12.440 (4) Å	µ = 0.12 mm^−^^1^
β = 101.833 (4)°	*T* = 120 K
*V* = 1732.6 (9) Å^3^	Plate, colorless
*Z* = 4	0.77 × 0.43 × 0.07 mm

### Data collection

**Table:**

Bruker D8 Advance single crystal CCD diffractometer	5594 independent reflections
Radiation source: fine-focus sealed tube	4796 reflections with *I* > 2σ(*I*)
Graphite monochromator	*R*_int_ = 0.039
Detector resolution: 8.3 pixels mm^-1^	θ_max_ = 25.1°, θ_min_ = 1.7°
Sets of exposures each taken over 0.5° ω rotation scans	*h* = −13→13
Absorption correction: multi-scan (*SADABS*; Bruker, 2014)	*k* = −15→12
*T*_min_ = 0.643, *T*_max_ = 1.000	*l* = −14→14
10435 measured reflections	

### Refinement

**Table:**

Refinement on *F*^2^	39 restraints
Least-squares matrix: full	Hydrogen site location: inferred from neighbouring sites
*R*[*F*^2^ > 2σ(*F*^2^)] = 0.041	H atoms treated by a mixture of independent and constrained refinement
*wR*(*F*^2^) = 0.095	*w* = 1/[σ^2^(*F*_o_^2^) + (0.0401*P*)^2^ + 0.0691*P*] where *P* = (*F*_o_^2^ + 2*F*_c_^2^)/3
*S* = 1.03	(Δ/σ)_max_ < 0.001
5594 reflections	Δρ_max_ = 0.27 e Å^−^^3^
454 parameters	Δρ_min_ = −0.18 e Å^−^^3^

### Special details

**Table:**

Geometry. All esds (except the esd in the dihedral angle between two l.s. planes) are estimated using the full covariance matrix. The cell esds are taken into account individually in the estimation of esds in distances, angles and torsion angles; correlations between esds in cell parameters are only used when they are defined by crystal symmetry. An approximate (isotropic) treatment of cell esds is used for estimating esds involving l.s. planes.
Refinement. One of the solvent molecules is disordered over two positions.

### Fractional atomic coordinates and isotropic or equivalent isotropic displacement parameters (Å^2^)

**Table:**

	*x*	*y*	*z*	*U*_iso_*/*U*_eq_	Occ. (<1)
O1A	0.0423 (2)	0.6609 (2)	0.3719 (2)	0.0280 (6)	
O2A	0.0083 (2)	0.3213 (2)	0.4273 (2)	0.0325 (7)	
O3A	−0.1629 (2)	0.3989 (2)	0.3414 (2)	0.0305 (7)	
N1A	0.2285 (3)	0.7482 (2)	0.5048 (2)	0.0199 (7)	
H1A	0.2165	0.7717	0.5708	0.030*	
H2A	0.1656	0.7711	0.4505	0.030*	
H3A	0.3028	0.7718	0.4929	0.030*	
N2A	0.1323 (2)	0.5031 (2)	0.3755 (2)	0.0190 (7)	
H4A	0.2002	0.4671	0.4009	0.023*	
C1A	0.2293 (3)	0.6338 (3)	0.5053 (3)	0.0183 (8)	
H11A	0.3123	0.6076	0.4960	0.022*	
C2A	0.1980 (3)	0.5909 (3)	0.6115 (3)	0.0222 (8)	
H21A	0.1192	0.6229	0.6220	0.027*	
H22A	0.1826	0.5161	0.6018	0.027*	
C3A	0.2968 (3)	0.6072 (3)	0.7160 (3)	0.0225 (8)	
H31A	0.3203	0.6816	0.7215	0.027*	
C4A	0.4133 (3)	0.5437 (3)	0.7136 (3)	0.0307 (10)	
H41A	0.4465	0.5625	0.6489	0.046*	
H42A	0.3919	0.4704	0.7103	0.046*	
H43A	0.4764	0.5573	0.7802	0.046*	
C5A	0.2433 (4)	0.5779 (4)	0.8157 (3)	0.0376 (11)	
H51A	0.1661	0.6164	0.8144	0.056*	
H52A	0.3040	0.5945	0.8830	0.056*	
H53A	0.2255	0.5040	0.8138	0.056*	
C6A	0.1269 (3)	0.5994 (3)	0.4086 (3)	0.0192 (8)	
C7A	0.0299 (3)	0.4538 (3)	0.2983 (3)	0.0180 (8)	
H71A	−0.0258	0.5090	0.2592	0.022*	
C8A	0.0799 (3)	0.3913 (3)	0.2128 (3)	0.0207 (8)	
H81A	0.1408	0.3403	0.2533	0.025*	
C9A	0.1501 (3)	0.4587 (3)	0.1444 (3)	0.0270 (9)	
H91A	0.2233	0.4895	0.1941	0.032*	
H92A	0.1820	0.4141	0.0918	0.032*	
C10A	0.0738 (4)	0.5449 (4)	0.0804 (3)	0.0442 (12)	
H12A	0.1226	0.5782	0.0324	0.066*	
H13A	0.0525	0.5957	0.1319	0.066*	
H14A	−0.0030	0.5165	0.0358	0.066*	
C11A	−0.0252 (3)	0.3306 (4)	0.1401 (3)	0.0350 (10)	
H15A	−0.0647	0.2854	0.1861	0.052*	
H16A	0.0093	0.2890	0.0877	0.052*	
H17A	−0.0873	0.3785	0.1000	0.052*	
C12A	−0.0472 (3)	0.3866 (3)	0.3615 (3)	0.0215 (8)	
O1B	0.5502 (2)	0.6220 (2)	0.50128 (19)	0.0238 (6)	
O2B	0.5573 (2)	0.3479 (2)	0.52421 (19)	0.0215 (6)	
O3B	0.3600 (2)	0.3917 (2)	0.45792 (19)	0.0216 (6)	
N1B	0.7727 (3)	0.7368 (2)	0.4733 (2)	0.0226 (7)	
H1B	0.8461	0.7665	0.5066	0.034*	
H2B	0.7080	0.7715	0.4921	0.034*	
H3B	0.7655	0.7392	0.3991	0.034*	
N2B	0.6296 (2)	0.5066 (2)	0.3983 (2)	0.0192 (7)	
H4B	0.6958	0.4877	0.3732	0.023*	
C1B	0.7706 (3)	0.6274 (3)	0.5093 (3)	0.0193 (8)	
H11B	0.8320	0.5861	0.4779	0.023*	
C2B	0.8047 (3)	0.6239 (3)	0.6345 (3)	0.0227 (8)	
H21B	0.8894	0.6530	0.6585	0.027*	
H22B	0.7462	0.6691	0.6636	0.027*	
C3B	0.8024 (3)	0.5183 (3)	0.6864 (3)	0.0248 (9)	
H31B	0.7208	0.4848	0.6546	0.030*	
C4B	0.8116 (4)	0.5314 (4)	0.8093 (3)	0.0371 (11)	
H41B	0.8115	0.4636	0.8438	0.056*	
H42B	0.7401	0.5715	0.8221	0.056*	
H43B	0.8891	0.5676	0.8411	0.056*	
C5B	0.9073 (4)	0.4492 (3)	0.6641 (3)	0.0343 (10)	
H51B	0.9013	0.3815	0.6974	0.052*	
H52B	0.9881	0.4804	0.6960	0.052*	
H53B	0.8997	0.4414	0.5847	0.052*	
C6B	0.6391 (3)	0.5850 (3)	0.4685 (3)	0.0189 (8)	
C7B	0.5130 (3)	0.4513 (3)	0.3620 (3)	0.0184 (8)	
H71B	0.4463	0.5021	0.3316	0.022*	
C8B	0.5294 (3)	0.3774 (3)	0.2694 (3)	0.0197 (8)	
H81B	0.6128	0.3439	0.2930	0.024*	
C9B	0.5323 (3)	0.4366 (3)	0.1635 (3)	0.0261 (9)	
H91B	0.4456	0.4501	0.1243	0.031*	
H92B	0.5735	0.5038	0.1826	0.031*	
C10B	0.6011 (4)	0.3791 (4)	0.0867 (3)	0.0393 (11)	
H12B	0.5968	0.4190	0.0192	0.059*	
H13B	0.5620	0.3118	0.0685	0.059*	
H14B	0.6886	0.3696	0.1231	0.059*	
C11B	0.4328 (3)	0.2917 (3)	0.2499 (3)	0.0249 (9)	
H15B	0.4419	0.2489	0.3159	0.037*	
H16B	0.4453	0.2493	0.1879	0.037*	
H17B	0.3489	0.3217	0.2332	0.037*	
C12B	0.4741 (3)	0.3938 (3)	0.4562 (3)	0.0175 (8)	
O1C	0.5265 (2)	0.7674 (2)	0.3049 (2)	0.0299 (7)	
H1C	0.501 (4)	0.784 (3)	0.364 (3)	0.045*	
F1C	0.2472 (2)	0.77562 (19)	0.09991 (19)	0.0419 (6)	
F2C	0.3722 (2)	0.64892 (18)	0.1427 (2)	0.0478 (7)	
F3C	0.2751 (2)	0.7092 (2)	0.26117 (19)	0.0454 (7)	
C1C	0.4374 (3)	0.8097 (3)	0.2199 (3)	0.0257 (9)	
H11C	0.4774	0.8281	0.1580	0.031*	
H12C	0.4032	0.8734	0.2459	0.031*	
C2C	0.3341 (3)	0.7359 (3)	0.1812 (3)	0.0285 (9)	
O1D	0.8241 (3)	0.7359 (3)	0.2588 (3)	0.0329 (10)	0.825 (5)
H1D	0.897 (4)	0.710 (4)	0.279 (4)	0.049*	0.825 (5)
F1D	0.6835 (3)	0.8066 (3)	0.0612 (3)	0.0441 (9)	0.825 (5)
F2D	0.8335 (3)	0.7120 (3)	0.0332 (3)	0.0576 (12)	0.825 (5)
F3D	0.6481 (3)	0.6516 (3)	0.0016 (2)	0.0572 (12)	0.825 (5)
C1D	0.7461 (4)	0.6710 (4)	0.1859 (3)	0.0280 (13)	0.825 (5)
H11D	0.6640	0.6662	0.2072	0.034*	0.825 (5)
H12D	0.7829	0.6012	0.1901	0.034*	0.825 (5)
C2D	0.7286 (4)	0.7099 (4)	0.0713 (3)	0.0353 (14)	0.825 (5)
O11D	0.8098 (19)	0.6853 (16)	0.2667 (12)	0.0329 (10)	0.175 (5)
H11E	0.8867	0.6965	0.2746	0.049*	0.175 (5)
F11D	0.7114 (17)	0.8328 (14)	0.1014 (19)	0.090 (11)*	0.175 (5)
F12D	0.8965 (12)	0.7813 (13)	0.1012 (14)	0.077 (6)*	0.175 (5)
F13D	0.7446 (19)	0.7253 (17)	−0.0213 (10)	0.116 (10)*	0.175 (5)
C11D	0.760 (2)	0.6624 (12)	0.1569 (13)	0.0280 (13)	0.175 (5)
H13D	0.6694	0.6480	0.1477	0.034*	0.175 (5)
H14D	0.8006	0.6000	0.1351	0.034*	0.175 (5)
C12D	0.7788 (13)	0.7499 (11)	0.0850 (10)	0.038 (8)*	0.175 (5)

### Atomic displacement parameters (Å^2^)

**Table:**

	*U*^11^	*U*^22^	*U*^33^	*U*^12^	*U*^13^	*U*^23^
O1A	0.0237 (14)	0.0214 (16)	0.0342 (15)	0.0061 (12)	−0.0051 (11)	−0.0037 (12)
O2A	0.0238 (13)	0.0317 (17)	0.0442 (17)	0.0035 (13)	0.0125 (12)	0.0137 (15)
O3A	0.0144 (13)	0.0460 (19)	0.0315 (15)	0.0020 (12)	0.0056 (11)	−0.0025 (14)
N1A	0.0179 (15)	0.0213 (19)	0.0198 (15)	−0.0020 (13)	0.0025 (12)	−0.0007 (13)
N2A	0.0143 (14)	0.0186 (19)	0.0224 (16)	0.0012 (12)	−0.0003 (12)	−0.0011 (14)
C1A	0.0182 (17)	0.014 (2)	0.0214 (19)	0.0003 (14)	0.0022 (14)	−0.0015 (15)
C2A	0.0227 (18)	0.021 (2)	0.023 (2)	−0.0005 (16)	0.0049 (15)	0.0016 (17)
C3A	0.030 (2)	0.017 (2)	0.0181 (18)	−0.0010 (16)	0.0003 (15)	−0.0002 (16)
C4A	0.026 (2)	0.040 (3)	0.024 (2)	0.0019 (19)	0.0003 (16)	0.0031 (19)
C5A	0.042 (2)	0.046 (3)	0.025 (2)	0.008 (2)	0.0083 (19)	0.003 (2)
C6A	0.0159 (17)	0.020 (2)	0.0212 (19)	−0.0007 (15)	0.0033 (14)	−0.0014 (16)
C7A	0.0162 (16)	0.018 (2)	0.0188 (18)	−0.0002 (15)	0.0007 (13)	0.0008 (15)
C8A	0.0222 (17)	0.021 (2)	0.0206 (19)	0.0013 (16)	0.0073 (14)	−0.0026 (17)
C9A	0.0266 (19)	0.032 (3)	0.023 (2)	−0.0011 (17)	0.0055 (15)	−0.0006 (18)
C10A	0.037 (2)	0.058 (3)	0.034 (3)	−0.003 (2)	0.000 (2)	0.018 (2)
C11A	0.031 (2)	0.038 (3)	0.038 (2)	−0.0091 (19)	0.0136 (19)	−0.014 (2)
C12A	0.0215 (19)	0.022 (2)	0.0221 (19)	0.0014 (17)	0.0079 (14)	−0.0087 (18)
O1B	0.0221 (13)	0.0259 (16)	0.0240 (14)	0.0017 (11)	0.0064 (11)	−0.0043 (12)
O2B	0.0196 (12)	0.0230 (16)	0.0219 (13)	0.0017 (10)	0.0044 (11)	0.0019 (11)
O3B	0.0152 (12)	0.0232 (15)	0.0265 (13)	0.0016 (11)	0.0048 (10)	0.0023 (12)
N1B	0.0214 (15)	0.0222 (19)	0.0251 (17)	−0.0002 (13)	0.0070 (13)	−0.0002 (14)
N2B	0.0169 (14)	0.0201 (18)	0.0213 (16)	0.0002 (12)	0.0060 (12)	−0.0024 (14)
C1B	0.0221 (18)	0.014 (2)	0.0229 (19)	−0.0009 (15)	0.0075 (15)	−0.0016 (16)
C2B	0.0206 (18)	0.023 (2)	0.0233 (19)	−0.0019 (15)	0.0026 (15)	−0.0022 (17)
C3B	0.0213 (18)	0.029 (2)	0.022 (2)	−0.0048 (16)	0.0010 (15)	0.0006 (17)
C4B	0.042 (2)	0.040 (3)	0.027 (2)	−0.006 (2)	0.0026 (19)	0.001 (2)
C5B	0.040 (2)	0.025 (3)	0.037 (2)	0.000 (2)	0.0062 (18)	0.003 (2)
C6B	0.0189 (17)	0.020 (2)	0.0174 (18)	0.0003 (15)	0.0037 (14)	0.0050 (16)
C7B	0.0146 (16)	0.019 (2)	0.0211 (19)	0.0007 (15)	0.0010 (14)	0.0012 (16)
C8B	0.0165 (17)	0.022 (2)	0.0202 (19)	−0.0003 (15)	0.0024 (14)	−0.0034 (16)
C9B	0.029 (2)	0.029 (2)	0.0203 (19)	−0.0030 (18)	0.0060 (16)	0.0003 (17)
C10B	0.038 (2)	0.059 (3)	0.024 (2)	−0.004 (2)	0.0127 (17)	−0.005 (2)
C11B	0.0212 (18)	0.028 (3)	0.024 (2)	−0.0013 (17)	0.0014 (15)	−0.0021 (17)
C12B	0.0185 (18)	0.013 (2)	0.0196 (18)	0.0022 (15)	0.0011 (14)	−0.0045 (16)
O1C	0.0289 (14)	0.0399 (19)	0.0208 (14)	0.0083 (13)	0.0045 (11)	0.0017 (13)
F1C	0.0378 (13)	0.0410 (17)	0.0400 (14)	−0.0028 (12)	−0.0079 (11)	0.0016 (12)
F2C	0.0526 (15)	0.0330 (17)	0.0551 (16)	0.0018 (12)	0.0048 (12)	−0.0159 (13)
F3C	0.0418 (14)	0.0557 (19)	0.0410 (15)	−0.0157 (12)	0.0140 (12)	0.0048 (13)
C1C	0.028 (2)	0.026 (2)	0.022 (2)	0.0010 (17)	0.0035 (16)	0.0003 (17)
C2C	0.035 (2)	0.026 (3)	0.024 (2)	0.0008 (18)	0.0042 (18)	0.0010 (18)
O1D	0.0277 (17)	0.039 (3)	0.0311 (17)	0.003 (2)	0.0032 (14)	0.004 (2)
F1D	0.054 (2)	0.041 (2)	0.035 (2)	0.0164 (17)	0.0036 (17)	0.0132 (18)
F2D	0.055 (2)	0.084 (3)	0.044 (2)	0.0129 (19)	0.0334 (17)	0.0123 (19)
F3D	0.073 (2)	0.062 (3)	0.0291 (17)	−0.0009 (19)	−0.0069 (16)	−0.0075 (16)
C1D	0.026 (2)	0.039 (3)	0.020 (3)	0.007 (2)	0.007 (2)	0.002 (2)
C2D	0.034 (3)	0.045 (4)	0.028 (3)	0.007 (3)	0.007 (2)	0.003 (3)
O11D	0.0277 (17)	0.039 (3)	0.0311 (17)	0.003 (2)	0.0032 (14)	0.004 (2)
C11D	0.026 (2)	0.039 (3)	0.020 (3)	0.007 (2)	0.007 (2)	0.002 (2)

### Geometric parameters (Å, º)

**Table:**

O1A—C6A	1.238 (4)	C1B—H11B	1.0000
O2A—C12A	1.247 (5)	C2B—C3B	1.519 (5)
O3A—C12A	1.251 (4)	C2B—H21B	0.9900
N1A—C1A	1.488 (5)	C2B—H22B	0.9900
N1A—H1A	0.9100	C3B—C4B	1.520 (5)
N1A—H2A	0.9100	C3B—C5B	1.526 (5)
N1A—H3A	0.9100	C3B—H31B	1.0000
N2A—C6A	1.323 (5)	C4B—H41B	0.9800
N2A—C7A	1.466 (4)	C4B—H42B	0.9800
N2A—H4A	0.8800	C4B—H43B	0.9800
C1A—C6A	1.533 (5)	C5B—H51B	0.9800
C1A—C2A	1.536 (5)	C5B—H52B	0.9800
C1A—H11A	1.0000	C5B—H53B	0.9800
C2A—C3A	1.525 (5)	C7B—C12B	1.523 (5)
C2A—H21A	0.9900	C7B—C8B	1.538 (5)
C2A—H22A	0.9900	C7B—H71B	1.0000
C3A—C4A	1.525 (5)	C8B—C11B	1.521 (5)
C3A—C5A	1.525 (5)	C8B—C9B	1.532 (5)
C3A—H31A	1.0000	C8B—H81B	1.0000
C4A—H41A	0.9800	C9B—C10B	1.527 (5)
C4A—H42A	0.9800	C9B—H91B	0.9900
C4A—H43A	0.9800	C9B—H92B	0.9900
C5A—H51A	0.9800	C10B—H12B	0.9800
C5A—H52A	0.9800	C10B—H13B	0.9800
C5A—H53A	0.9800	C10B—H14B	0.9800
C7A—C8A	1.526 (5)	C11B—H15B	0.9800
C7A—C12A	1.538 (5)	C11B—H16B	0.9800
C7A—H71A	1.0000	C11B—H17B	0.9800
C8A—C11A	1.528 (5)	O1C—C1C	1.396 (4)
C8A—C9A	1.534 (5)	O1C—H1C	0.86 (3)
C8A—H81A	1.0000	F1C—C2C	1.342 (4)
C9A—C10A	1.522 (6)	F2C—C2C	1.328 (4)
C9A—H91A	0.9900	F3C—C2C	1.338 (4)
C9A—H92A	0.9900	C1C—C2C	1.486 (5)
C10A—H12A	0.9800	C1C—H11C	0.9900
C10A—H13A	0.9800	C1C—H12C	0.9900
C10A—H14A	0.9800	O1D—C1D	1.394 (4)
C11A—H15A	0.9800	O1D—H1D	0.86 (3)
C11A—H16A	0.9800	F1D—C2D	1.347 (4)
C11A—H17A	0.9800	F2D—C2D	1.329 (4)
O1B—C6B	1.228 (4)	F3D—C2D	1.338 (5)
O2B—C12B	1.259 (4)	C1D—C2D	1.487 (5)
O3B—C12B	1.254 (4)	C1D—H11D	0.9900
N1B—C1B	1.493 (5)	C1D—H12D	0.9900
N1B—H1B	0.9100	O11D—C11D	1.396 (6)
N1B—H2B	0.9100	O11D—H11E	0.8400
N1B—H3B	0.9100	F11D—C12D	1.345 (6)
N2B—C6B	1.333 (5)	F12D—C12D	1.327 (6)
N2B—C7B	1.453 (4)	F13D—C12D	1.337 (6)
N2B—H4B	0.8800	C11D—C12D	1.488 (6)
C1B—C2B	1.526 (5)	C11D—H13D	0.9900
C1B—C6B	1.528 (5)	C11D—H14D	0.9900
			
C1A—N1A—H1A	109.5	H21B—C2B—H22B	107.4
C1A—N1A—H2A	109.5	C2B—C3B—C4B	108.8 (3)
H1A—N1A—H2A	109.5	C2B—C3B—C5B	112.1 (3)
C1A—N1A—H3A	109.5	C4B—C3B—C5B	110.6 (3)
H1A—N1A—H3A	109.5	C2B—C3B—H31B	108.4
H2A—N1A—H3A	109.5	C4B—C3B—H31B	108.4
C6A—N2A—C7A	122.7 (3)	C5B—C3B—H31B	108.4
C6A—N2A—H4A	118.7	C3B—C4B—H41B	109.5
C7A—N2A—H4A	118.7	C3B—C4B—H42B	109.5
N1A—C1A—C6A	106.6 (3)	H41B—C4B—H42B	109.5
N1A—C1A—C2A	111.4 (3)	C3B—C4B—H43B	109.5
C6A—C1A—C2A	108.2 (3)	H41B—C4B—H43B	109.5
N1A—C1A—H11A	110.2	H42B—C4B—H43B	109.5
C6A—C1A—H11A	110.2	C3B—C5B—H51B	109.5
C2A—C1A—H11A	110.2	C3B—C5B—H52B	109.5
C3A—C2A—C1A	116.0 (3)	H51B—C5B—H52B	109.5
C3A—C2A—H21A	108.3	C3B—C5B—H53B	109.5
C1A—C2A—H21A	108.3	H51B—C5B—H53B	109.5
C3A—C2A—H22A	108.3	H52B—C5B—H53B	109.5
C1A—C2A—H22A	108.3	O1B—C6B—N2B	123.9 (3)
H21A—C2A—H22A	107.4	O1B—C6B—C1B	120.3 (3)
C4A—C3A—C5A	110.0 (3)	N2B—C6B—C1B	115.8 (3)
C4A—C3A—C2A	111.0 (3)	N2B—C7B—C12B	111.7 (3)
C5A—C3A—C2A	109.6 (3)	N2B—C7B—C8B	108.2 (3)
C4A—C3A—H31A	108.7	C12B—C7B—C8B	111.3 (3)
C5A—C3A—H31A	108.7	N2B—C7B—H71B	108.5
C2A—C3A—H31A	108.7	C12B—C7B—H71B	108.5
C3A—C4A—H41A	109.5	C8B—C7B—H71B	108.5
C3A—C4A—H42A	109.5	C11B—C8B—C9B	111.5 (3)
H41A—C4A—H42A	109.5	C11B—C8B—C7B	113.2 (3)
C3A—C4A—H43A	109.5	C9B—C8B—C7B	110.9 (3)
H41A—C4A—H43A	109.5	C11B—C8B—H81B	107.0
H42A—C4A—H43A	109.5	C9B—C8B—H81B	107.0
C3A—C5A—H51A	109.5	C7B—C8B—H81B	107.0
C3A—C5A—H52A	109.5	C10B—C9B—C8B	113.1 (3)
H51A—C5A—H52A	109.5	C10B—C9B—H91B	109.0
C3A—C5A—H53A	109.5	C8B—C9B—H91B	109.0
H51A—C5A—H53A	109.5	C10B—C9B—H92B	109.0
H52A—C5A—H53A	109.5	C8B—C9B—H92B	109.0
O1A—C6A—N2A	125.0 (3)	H91B—C9B—H92B	107.8
O1A—C6A—C1A	118.2 (3)	C9B—C10B—H12B	109.5
N2A—C6A—C1A	116.6 (3)	C9B—C10B—H13B	109.5
N2A—C7A—C8A	110.7 (3)	H12B—C10B—H13B	109.5
N2A—C7A—C12A	109.8 (3)	C9B—C10B—H14B	109.5
C8A—C7A—C12A	111.5 (3)	H12B—C10B—H14B	109.5
N2A—C7A—H71A	108.3	H13B—C10B—H14B	109.5
C8A—C7A—H71A	108.3	C8B—C11B—H15B	109.5
C12A—C7A—H71A	108.3	C8B—C11B—H16B	109.5
C7A—C8A—C11A	110.7 (3)	H15B—C11B—H16B	109.5
C7A—C8A—C9A	112.0 (3)	C8B—C11B—H17B	109.5
C11A—C8A—C9A	111.6 (3)	H15B—C11B—H17B	109.5
C7A—C8A—H81A	107.4	H16B—C11B—H17B	109.5
C11A—C8A—H81A	107.4	O3B—C12B—O2B	124.3 (3)
C9A—C8A—H81A	107.4	O3B—C12B—C7B	117.4 (3)
C10A—C9A—C8A	115.3 (3)	O2B—C12B—C7B	118.2 (3)
C10A—C9A—H91A	108.5	C1C—O1C—H1C	104 (3)
C8A—C9A—H91A	108.5	O1C—C1C—C2C	111.0 (3)
C10A—C9A—H92A	108.5	O1C—C1C—H11C	109.4
C8A—C9A—H92A	108.5	C2C—C1C—H11C	109.4
H91A—C9A—H92A	107.5	O1C—C1C—H12C	109.4
C9A—C10A—H12A	109.5	C2C—C1C—H12C	109.4
C9A—C10A—H13A	109.5	H11C—C1C—H12C	108.0
H12A—C10A—H13A	109.5	F2C—C2C—F3C	106.4 (3)
C9A—C10A—H14A	109.5	F2C—C2C—F1C	106.4 (3)
H12A—C10A—H14A	109.5	F3C—C2C—F1C	106.5 (3)
H13A—C10A—H14A	109.5	F2C—C2C—C1C	113.1 (3)
C8A—C11A—H15A	109.5	F3C—C2C—C1C	112.2 (3)
C8A—C11A—H16A	109.5	F1C—C2C—C1C	111.7 (3)
H15A—C11A—H16A	109.5	C1D—O1D—H1D	111 (4)
C8A—C11A—H17A	109.5	O1D—C1D—C2D	111.0 (4)
H15A—C11A—H17A	109.5	O1D—C1D—H11D	109.4
H16A—C11A—H17A	109.5	C2D—C1D—H11D	109.4
O2A—C12A—O3A	123.8 (3)	O1D—C1D—H12D	109.4
O2A—C12A—C7A	118.6 (3)	C2D—C1D—H12D	109.4
O3A—C12A—C7A	117.6 (3)	H11D—C1D—H12D	108.0
C1B—N1B—H1B	109.5	F2D—C2D—F3D	106.4 (3)
C1B—N1B—H2B	109.5	F2D—C2D—F1D	106.0 (4)
H1B—N1B—H2B	109.5	F3D—C2D—F1D	106.7 (4)
C1B—N1B—H3B	109.5	F2D—C2D—C1D	113.4 (4)
H1B—N1B—H3B	109.5	F3D—C2D—C1D	111.5 (4)
H2B—N1B—H3B	109.5	F1D—C2D—C1D	112.3 (3)
C6B—N2B—C7B	121.8 (3)	C11D—O11D—H11E	109.5
C6B—N2B—H4B	119.1	O11D—C11D—C12D	110.5 (7)
C7B—N2B—H4B	119.1	O11D—C11D—H13D	109.6
N1B—C1B—C2B	108.6 (3)	C12D—C11D—H13D	109.6
N1B—C1B—C6B	108.4 (3)	O11D—C11D—H14D	109.6
C2B—C1B—C6B	110.2 (3)	C12D—C11D—H14D	109.6
N1B—C1B—H11B	109.9	H13D—C11D—H14D	108.1
C2B—C1B—H11B	109.9	F12D—C12D—F13D	106.9 (7)
C6B—C1B—H11B	109.9	F12D—C12D—F11D	106.0 (7)
C3B—C2B—C1B	116.0 (3)	F13D—C12D—F11D	106.8 (7)
C3B—C2B—H21B	108.3	F12D—C12D—C11D	113.3 (6)
C1B—C2B—H21B	108.3	F13D—C12D—C11D	111.5 (7)
C3B—C2B—H22B	108.3	F11D—C12D—C11D	111.9 (6)
C1B—C2B—H22B	108.3		
			
N1A—C1A—C2A—C3A	−69.5 (4)	C7B—N2B—C6B—C1B	173.1 (3)
C6A—C1A—C2A—C3A	173.6 (3)	N1B—C1B—C6B—O1B	−63.1 (4)
C1A—C2A—C3A—C4A	−68.2 (4)	C2B—C1B—C6B—O1B	55.6 (4)
C1A—C2A—C3A—C5A	170.1 (3)	N1B—C1B—C6B—N2B	117.8 (3)
C7A—N2A—C6A—O1A	−6.9 (5)	C2B—C1B—C6B—N2B	−123.5 (3)
C7A—N2A—C6A—C1A	168.6 (3)	C6B—N2B—C7B—C12B	−65.5 (4)
N1A—C1A—C6A—O1A	−21.6 (4)	C6B—N2B—C7B—C8B	171.7 (3)
C2A—C1A—C6A—O1A	98.4 (4)	N2B—C7B—C8B—C11B	161.1 (3)
N1A—C1A—C6A—N2A	162.6 (3)	C12B—C7B—C8B—C11B	38.0 (4)
C2A—C1A—C6A—N2A	−77.5 (4)	N2B—C7B—C8B—C9B	−72.7 (3)
C6A—N2A—C7A—C8A	136.9 (3)	C12B—C7B—C8B—C9B	164.2 (3)
C6A—N2A—C7A—C12A	−99.6 (4)	C11B—C8B—C9B—C10B	−77.4 (4)
N2A—C7A—C8A—C11A	173.8 (3)	C7B—C8B—C9B—C10B	155.5 (3)
C12A—C7A—C8A—C11A	51.3 (4)	N2B—C7B—C12B—O3B	142.0 (3)
N2A—C7A—C8A—C9A	−60.9 (4)	C8B—C7B—C12B—O3B	−97.0 (4)
C12A—C7A—C8A—C9A	176.5 (3)	N2B—C7B—C12B—O2B	−41.1 (4)
C7A—C8A—C9A—C10A	−59.5 (4)	C8B—C7B—C12B—O2B	80.0 (4)
C11A—C8A—C9A—C10A	65.2 (4)	O1C—C1C—C2C—F2C	−60.4 (4)
N2A—C7A—C12A—O2A	−52.8 (4)	O1C—C1C—C2C—F3C	60.0 (4)
C8A—C7A—C12A—O2A	70.2 (4)	O1C—C1C—C2C—F1C	179.6 (3)
N2A—C7A—C12A—O3A	129.1 (3)	O1D—C1D—C2D—F2D	63.6 (5)
C8A—C7A—C12A—O3A	−107.9 (4)	O1D—C1D—C2D—F3D	−176.3 (4)
N1B—C1B—C2B—C3B	177.8 (3)	O1D—C1D—C2D—F1D	−56.6 (5)
C6B—C1B—C2B—C3B	59.2 (4)	O11D—C11D—C12D—F12D	52.3 (19)
C1B—C2B—C3B—C4B	−168.3 (3)	O11D—C11D—C12D—F13D	172.9 (18)
C1B—C2B—C3B—C5B	69.1 (4)	O11D—C11D—C12D—F11D	−67.5 (19)
C7B—N2B—C6B—O1B	−5.9 (5)		

### Hydrogen-bond geometry (Å, º)

**Table:**

*D*—H···*A*	*D*—H	H···*A*	*D*···*A*	*D*—H···*A*
N1*A*—H1*A*···O3*A*^i^	0.91	2.13	2.928 (4)	146
N1*A*—H2*A*···O1*A*	0.91	2.07	2.607 (4)	116
N1*A*—H3*A*···O2*B*^ii^	0.91	1.87	2.767 (4)	168
N2*A*—H4*A*···O3*B*	0.88	2.00	2.883 (4)	177
N1*B*—H1*B*···O2*A*^ii^	0.91	1.79	2.695 (4)	179
N1*B*—H2*B*···O3*B*^ii^	0.91	1.89	2.721 (4)	151
N1*B*—H3*B*···O1*D*	0.91	1.98	2.838 (5)	156
O1*D*—H1*D*···O1*A*^iii^	0.86 (3)	1.87 (4)	2.695 (4)	159 (5)
O1*C*—H1*C*···O2*B*^ii^	0.86 (3)	1.85 (3)	2.693 (4)	167 (4)

Symmetry codes: (i) −*x*, *y*+1/2, −*z*+1; (ii) −*x*+1, *y*+1/2, −*z*+1; (iii) *x*+1, *y*, *z*.
